# Genome sequence of *Stenotrophomonas geniculata* AS50Mn isolated from sand filters used to treat manganese-containing alkaline mine water

**DOI:** 10.1128/mra.01252-25

**Published:** 2026-02-27

**Authors:** Hiroshi Habe, Tomohiro Inaba

**Affiliations:** 1 Environmental Management Research Institute, National Institute of Advanced Industrial Science and Technology 73461 , Tsukuba, Ibaraki, Japan; Wellesley College, Wellesley, Massachusetts, USA

**Keywords:** alkaline-tolerant bacteria, manganese-containing mine water, manganese dioxide-coated sand, rapid sand filter, *Stenotrophomonas geniculata*

## Abstract

*Stenotrophomonas geniculata* AS50Mn was isolated from sand filters used to treat manganese-containing mine water under alkaline conditions. Here, we report the complete genome sequence of this bacterium, which comprises a 4,478,846 bp circular chromosome. The genome contains 4,082 coding DNA sequences, 13 rRNA genes, and 79 tRNA genes.

## ANNOUNCEMENT

Even after mine closure, continuous chemical treatment of mine water containing metals and metalloids is required (1). A pilot-scale rapid sand filter system was applied to treat manganese (Mn)-containing mine water at a mining site. When the pH of the inlet water was adjusted to 8.5–9.0, Mn removal rates reached 89.5% and 95.0%, respectively (2). During this process, *Stenotrophomonas* sp. AS50Mn was isolated from the sand filter, where it was identified as the predominant species in the microbial community (2, 3). Sand filter samples were plated on 50K medium agar (pH 8.2), containing 9.5 g/L MnSO_4_·5H_2_O, 2.0 g/L Bacto Peptone, 0.5 g/L Bacto Yeast Extract, and 2.38 g/L (10 mM) HEPES. After several rounds of subculturing at 30°C, a single colony type was isolated, confirmed for purity, and identified as *Stenotrophomonas* (3). Strain AS50Mn demonstrated growth across Mn^2+^ concentrations ranging from 2.75 to 44 mg/L at pH 8.5 and up to 22 mg/L at pH 9.0 (3). However, the molecular basis underlying its resistance to alkaline pH and Mn^2+^ remains unclear.

A single colony of strain AS50Mn was picked from an R2A agar plate (Daigo; Shiotani M.S. Co., Ltd., Amagasaki, Japan), inoculated into 30 mL of the R2A broth liquid medium, and cultivated at 30°C for 24 h. Genomic DNA was extracted following a standard protocol (4). Genome sequencing was performed at the Taniguchi Dental Clinic Oral Microbiome Center (Kagawa, Japan) following standard library preparation workflows. For short-read sequencing, genomic libraries were prepared using the Illumina DNA Prep (M) Tagmentation Kit (Illumina, San Diego, CA, USA) according to the Kit protocol, and sequencing was performed on a MiSeq platform using the MiSeq Reagent Kit v2 (300 cycles, Illumina). For long-read sequencing, genomic libraries were prepared using the Native Barcode Sequencing Kit (SQK-NBD114-24; Oxford Nanopore Technologies, Oxford, UK) according to the Kit protocol after treatment with Short Read Eliminator XS (PacBio, Menlo Park, CA, USA) to remove <10 kb DNA, and sequenced using a PromethION 2i platform with FLO-PRO114M flow cells (Oxford Nanopore Technologies). The software used in the long-read sequence were as follows: MinKNOW v23.11.8, Bream v7.8.2, Configuration v5.8.6, Dorado v7.2.14, and MinKNOW Core v5.8.9.

A total of 500,296,458 paired-end reads (150 bp) were obtained from the Illumina platform (number of sequences: 3,285,102; *N*_50_: 152), and 1,269,072,355 single-end reads were obtained from the Nanopore platform (number of sequences: 204,812; *N*_50_: 6,196). Adapter and low-quality reads were trimmed using Fastp (5) v0.20.1 for Illumina data and NanoPlot (6) v1.32.1 and NanoFilt (7) v2.7.1 for Nanopore data. After quality filtering, a total of 1,221,569,407 trimmed reads were used for *de novo* assembly with Unicycler (8) v0.4.8 and Pilon (9) v1.24, applying standard evaluation strategies (10, 11). Circularity and replicon boundaries were determined using Flye (12) v2.9.1. Also, the assembly graph visualized in Bandage (13) v0.8.1 confirmed the genome to be circular. In Unicycler, the genome was rotated to start at the *dnaA* gene. Gene annotation was performed using DFAST (14) v1.6.0. Completeness was assessed using CheckM v1.1.6. Default parameters were applied for all software unless otherwise specified.

The genome consisted of 4,478,846 bp with a coverage of 111×, an average GC content of 66.5%, and 4,082 predicted coding sequences. The genome also contained 13 rRNA and 79 tRNA genes (Table 1). Based on genome BLAST distance phylogeny analysis, the genome of strain AS50Mn exhibited an average nucleotide identity of 95.9% with *S. geniculata* BR23 (accession number CP134450). According to the high similarity of whole genome sequences, strain AS50Mn was classified as *S. geniculata*. The transcriptomic analysis based on this genome may yield additional information regarding the bacterium’s survival and adaptation in sand filters used to treat manganese-rich alkaline mine water.

**TABLE 1:** Annotation statistics

	Value
Total sequence length (bp)	4,478,846
Number of sequences	1
Longest sequences (bp)	4,478,846
*N*_50_ (bp)	4,478,846
Gap ratio (%)	0
GC content (%)	66.5
Number of CDSs	4,082
Average protein length (aa)	327.6
Coding ratio (%)	89.6
Number of rRNAs	13
Number of tRNAs	79
Number of CRISPRs	2

## Data Availability

The genome sequence has been deposited in DDBJ/EMBL/GenBank under accession number BAAGIK010000001. The raw sequence data are available under accession numbers DRR627340 (Illumina) and DRR627341 (Nanopore). The BioProject is registered under accession number PRJDB19894.
